# Zika virus infection triggers lipophagy by stimulating the AMPK-ULK1 signaling in human hepatoma cells

**DOI:** 10.3389/fcimb.2022.959029

**Published:** 2022-11-02

**Authors:** Zhao-Ling Qin, Qiu-Feng Yao, Ping Zhao, Hao Ren, Zhong-Tian Qi

**Affiliations:** Department of Microbiology, Naval Medical University, Shanghai, China

**Keywords:** Zika virus, lipophagy, AMPK, ULK1, mTOR, lipid metabolism

## Abstract

Zika virus (ZIKV) is a globally transmitted mosquito-borne pathogen, and no effective treatment or vaccine is available yet. Lipophagy, a selective autophagy targeting lipid droplets (LDs), is an emerging subject in cellular lipid metabolism and energy homeostasis. However, the regulatory mechanism of lipid metabolism and the role of lipophagy in Zika virus infection remain largely unknown. Here, we demonstrated that ZIKV induced lipophagy by activating unc-51-like kinase 1 (ULK1) through activation of 5’ adenosine monophosphate (AMP)-activated protein kinase (AMPK) in Huh7 cells. Upon ZIKV infection, the average size and triglyceride content of LDs significantly decreased. Moreover, ZIKV infection significantly increased lysosomal biosynthesis and LD-lysosome fusion. The activities of AMPK at Thr-172 and ULK1 at Ser-556 were increased in ZIKV-infected cells and closely correlated with lipophagy induction. Silencing of AMPK expression inhibited ZIKV infection, autophagy induction, and LD-lysosome fusion and decreased the triglyceride content of the cells. The activities of mammalian target of rapamycin (mTOR) at Ser-2448 and ULK1 at Ser-757 were suppressed independently of AMPK during ZIKV infection. Therefore, ZIKV infection triggers AMPK-mediated lipophagy, and the LD-related lipid metabolism during ZIKV infection is mainly regulated *via* the AMPK-ULK1 signaling pathway.

## Introduction

Zika virus (ZIKV) is a mosquito-borne pathogen mainly transmitted by *Aedes* and has recently spread worldwide. Zika virus disease caused by ZIKV infection is an imminent threat to human health. Its clinical symptoms include fever, rash, conjunctivitis, and joint and muscle pain and are also associated with neurological complications, such as neonatal microcephaly and Guillain-Barré syndrome (Broutet et al., 2016; Calvet et al., 2016; Mlakar et al., 2016).

ZIKV belongs to the *Flavivirus* genus of the *Flaviviridae* family (Ledermann et al., 2014). The viral genome is a single-stranded ~11 kb positive RNA and contains a single open reading frame, which encodes three structural proteins (capsid [C], precursor membrane [prM], and envelope [E] proteins) and seven nonstructural (NS) proteins (NS1, NS2A, NS2B, NS3, NS4A, NS4B, and NS5 proteins) (Kuno et al., 1998). Various types of cells support ZIKV infection and replication effectively, but the molecular mechanisms underlying the early stages of ZIKV infection remain unclear. Thus, given that there is no vaccine against ZIKV infection, it is important to elucidate the interaction between ZIKV and host cells for the development of anti-viral therapies.

Autophagy is a conserved process that maintains cell homeostasis. Autophagosomes are formed from double-membrane vesicles in the cytoplasm and then encapsulate and sequester endogenous substrates, such as intracellular aged proteins and damaged organelles. Mature autophagosomes finally fuse with lysosomes to form autophagolysosomes, which in turn catabolize the contents, realize the reuse of intracellular substances and chemical energy, delay senescence, and maintain cell homeostasis (Mizushima, 2007). Robust autophagy also helps cells resist infection by pathogens such as herpes simplex virus 1 (HSV-1), vesicular stomatitis virus (VSV), and Sindbis virus (Orvedahl et al., 2007; Shelly et al., 2009). However, several viruses with a positive-stranded RNA genome have developed a strategy that utilizes the host autophagy element to efficiently replicate. These viruses include hepatitis C virus (HCV), dengue virus (DENV), and ZIKV (Dreux et al., 2009; Khakpoor et al., 2009; Heaton and Randall, 2010; Peng et al., 2018). After inhibition of autophagy, viral replication and release of viral progeny are significantly reduced, but the mechanism of how viruses utilize host autophagy to facilitate viral infection is not fully understood.

Autophagy is often considered a general and non-selective degradation process. However, increasing studies have indicated that substances to be degraded by autophagy are selectively delivered to the autophagy pathway through unique organelle-specific linkers (Kim et al., 2007; Kraft et al., 2008; Khaminets et al., 2015). Autophagy that specifically targets lipid droplets (LDs) is called lipophagy, which is shown to regulate cellular lipid metabolism (Singh et al., 2009). LDs have a unique structure consisting of a neutral lipid core and a surrounding monolayer phospholipid membrane. Although LDs serve as reservoirs of cellular triglycerides (TGs) and cholesteryl esters, they are also dynamic organelles that participate in various cellular metabolic processes and provide cells with important energy reserves (Martin and Parton, 2006; Farese and Walther, 2009). In addition to lipolysis and fat synthesis, lipophagy is regarded as a new and alternative mechanism to maintain LD homeostasis (Ward et al., 2016; Shin, 2020). The host factors involved in lipophagy regulation and their mechanistic roles in LD metabolism and energy homeostasis remain unknown. Reports on other flaviviruses imply that the regulatory mechanisms underlying lipophagy involve LD-related molecules and cellular signaling networks. The NS4A and NS4B proteins of DENV interact with the host LD-associated factor AUP1. They hijack the acyltransferase function of AUP1, thereby triggering the consumption of LDs, which in turn promotes the production of viral progeny (Zhang, J. S. et al., 2018). Dengue virus has been reported to induce 5’ adenosine monophosphate (AMP)-activated protein kinase (AMPK)-dependent lipophagy, mobilize lipid storage, and enhance β-oxidation, thereby promoting viral replication (Jordan and Randall, 2017). Sirtuin 3 (SIRT3) activates lipophagy by stimulating the AMPK-unc-51-like kinase 1 (ULK1) pathway and subsequently reduces LD size and lipid accumulation in mature adipocytes (Zhang et al., 2020).

Mammalian target of rapamycin (mTOR) is a classic upstream signaling node regulating autophagy. The functional mTOR complex 1 (mTORC1) is responsible for the regulation of protein synthesis and metabolism, cell growth, and autophagy inhibition (Laplante and Sabatini, 2012). Under nutrient-rich conditions, mTORC1 directly interacts with the ULK1-Atg13-FIP200 complex and phosphorylates ULK1 and Atg13, and consequently inhibits autophagy. Once mTORC1 is inactivated, ULK1 and Atg13 are dephosphorylated, thereby deactivating autophagy inhibition (Hosokawa et al., 2009). Additionally, mTORC1 phosphorylates transcription factor EB (TFEB), preventing its translocation to the nucleus from the cytoplasm, thereby inhibiting the transcription of autophagy- and lysosome-related genes (Martina et al., 2012).

AMPK is a sensor of cellular energy status and regulates autophagy by inhibiting the activity of mTORC1. AMPK is activated by phosphorylation at its threonine 172 residue and then phosphorylates tuberous sclerosis complex 2 (TSC2). Consequently, Rheb is inactivated, and mTORC1 activity is inhibited (Inoki et al., 2003). Additionally, AMPK can directly induce autophagy by phosphorylating the key autophagy complex, the ULK1/2 complex, independently of mTORC1 (Kim et al., 2013). AMPK is also a major factor whose activity is modulated by viruses to generate a favorable host lipid environment for their own replication. For example, human cytomegalovirus (HCMV) infection enhances AMPK activity to trigger glucose import and the glycolytic pathway, which is essential for HCMV replication (McArdle et al., 2012). DENV infection activates AMPK to facilitate the viral replication and induce lipophagy (Jordan and Randall, 2017). However, viral inhibition of AMPK activity to allow viral replication has also been shown. AMPK activity is decreased during early DENV infection. Inhibition of AMPK increases the HMGCR reductase activity, leading to cholesterol accumulation in replication sites to support DENV replication (Soto-Acosta et al., 2017). Inhibition of AMPK is accompanied by intracellular lipid accumulation during HCV infection (Mankouri et al., 2010).

Several studies have demonstrated that autophagy is induced by ZIKV infection in both *in vitro* and *in vivo* models (Liang et al., 2016; Chiramel and Best, 2018; Peng et al., 2018; Gratton et al., 2019; Tiwari et al., 2020). Like other flaviviruses, ZIKV can also invade the liver, which is especially rich in LDs, and consequently cause related liver dysfunction including hepatitis (Pol et al., 2014; Ferreira et al., 2018; Sherman et al., 2019). In this study, we examined lipophagy in ZIKV-infected Huh7 cells and also explored the roles of AMPK and its downstream ULK1 and mTOR signaling pathways during this process.

## Materials and methods

### Cells and virus

Human hepatoma *cells* (*Huh7*, kindly provided by Dr Jing Zhong, Institute Pasteur of Shanghai, Chinese Academy of Sciences, Shanghai, China) were grown in complete Dulbecco’s Modified Eagle Medium (DMEM) with 10% (v/v) heat-inactivated fetal bovine serum (FBS) (Gibco BRL, USA), 100 nM nonessential amino acids (NEAA) (Invitrogen, Shanghai, China), 1 mM L-glutamine, 100 μg streptomycin/ml, and 100 U penicillin/ml at 37 °C with 5% CO_2_. C6/36 cells (kindly provided by Professor Jing An from Capital Medical University, Beijing, China) were cultured in RPMI1640 (Gibco) with 10% FBS at 28 °C with 5% CO_2_. ZIKV (strain SZ01, kindly provided by Professor Cheng-Feng Qin from Beijing Institute of Microbiology and Epidemiology, Beijing, China) was propagated in C6/36 cells, and the stock virus was stored at -80 °C until use. Virus titers were determined using the plaque assay and expressed as plaque-forming unit (PFU) per milliliter.

### Antibodies and small interfering RNAs

Rabbit anti-ZIKV Capsid and NS3 polyclonal antibodies, and mouse anti-LAMP1 monoclonal antibody were purchased from GeneTex (Irvine, CA, USA). Rabbit anti-LC3B polyclonal antibody was purchased from Sigma Aldrich (St. Louis, MO, USA). Rabbit anti-GAPDH monoclonal antibody, rabbit anti-phospho-ULK1 (Ser556) polyclonal antibody, mouse anti-SCD1 monoclonal antibody, and rabbit anti-ACC1 monoclonal antibody were purchased from Abcam (Cambridge, MA, USA). Rabbit anti-AMPKα polyclonal antibody, rabbit anti-phospho-AMPKα (Thr172) monoclonal antibody, rabbit anti-mTOR polyclonal antibody, rabbit anti-phospho-mTOR (Ser2448) polyclonal antibody, rabbit anti-ATGL polyclonal antibody, and rabbit anti- phospho-HSL (Ser660) polyclonal antibody were purchased from Cell Signaling Technology (Massachusetts, USA). Rabbit anti-phospho-ULK1 (Ser757) and rabbit anti-FASN polyclonal antibodies were purchased from Abclonal (Wuhan, China). Rabbit anti-LAMP2a monoclonal antibody, rabbit anti-TFEB polyclonal antibody, and rabbit anti-ULK1 polyclonal antibody were purchased from Beyotime (Hangzhou, China). Rabbit anti-HSL polyclonal antibody, Alex Fluor 488-conjugated donkey anti-rabbit IgG, Alex Fluor 594-conjugated donkey anti-rabbit IgG, HRP-conjugated goat anti-rabbit IgG, and HRP-conjugated goat anti-mouse IgG were purchased from Thermo Fisher scientific. AMPKα siRNA (target sequence: 5’-ACAGGAGAATAATGAATGA-3’) and a scrambled negative control siRNA (siNC) were obtained from RiboBio (Guangzhou, China).

### Viral infection

Huh7 cells were infected with ZIKV at a multiplicity of infection (MOI) of 0.5 or 1 for 1 h in serum-free Opti-MEM. Subsequently, the cells were washed with PBS and then cultured in fresh complete medium for various periods until they were harvested and examined.

### siRNA transfection

siRNAs were introduced into Huh7 cells by using Lipofectamine 3000 (Thermo Fisher Scientific) according to the manufacturer’s instructions. Briefly, cells were seeded onto 24-well tissue culture plates (1 × 10^5^ cells/well) and grown overnight until 95% confluence. Next day, 25 nmol siRNA was mixed with 25 μl Opti-MEM for 5 min at room temperature (RT). The mixture was then added to 25 μl Opti-MEM with 1 μl Lipofectamine 3000 that had undergone similar incubation conditions. After being incubated for 15 min, the siRNA-liposome complexes were added onto the cells. Cells were incubated at 37°C for 48 h to ensure effective gene knockdown. Then the cells transfected with siRNAs were infected with ZIKV (MOI = 0.5 or 1) for 1 h and maintained for an additional 24 h at 37°C. After infection, the cells were subjected to western blot or immunofluorescence analyses.

### Oil Red O or BODIPY staining

To visualize LDs, treated Huh7 cells were washed twice with sterile PBS and fixed with 4% paraformaldehyde for 15 min at RT. After being washed three times with PBS, the cells were stained with freshly prepared ORO (Sigma, USA) or BODIPY 493/503 dye (Thermo Fisher Scientific, D3922) for 20 min at RT. Then, the staining solution was removed, and the samples were incubated with 4’, 6’- diamidino-2-phenylindole (DAPI, Roche, Switzerland) to stain the nuclei. Finally, the samples were washed with PBS for three times. The stained LDs were observed using confocal microscopy and then photographed.

### Immunofluorescence and confocal microscopy

Cell immunofluorescence assay was performed as previously described (Khakpooret al., 2009; Gao et al., 2015). Briefly, treated Huh7 cells were cultured on a chambered coverglass (Thermo Fisher Scientific) and then fixed with 4% paraformaldehyde for 15 min at RT. The samples were permeabilized with 0.1% Triton X-100 for 5 min at RT and then blocked with 3% BSA (in PBS) for 2 h. Afterward, they were incubated with the indicated primary antibodies (2 h at RT) and fluorophore-conjugated secondary antibodies (1 h at RT) in 3% BSA and then stained with DAPI. The samples were observed using a Zeiss LSM 800 confocal fluorescence microscope. Images were adjusted for brightness and contrast and collocated into figures by using Photoshop CS8.

### Quantitation of intracellular TG content

TGs were extracted from treated Huh7 cells and quantitated using a Triglyceride Assay Kit (Sigma, USA) according to the manufacturer’s instructions. Briefly, the lysate suspension was incubated at 90°C for 30 min, and then its TG level was measured *via* an enzymatic method. The amount of TGs in the samples was calculated according to the standard curve obtained from the TG standards provided by the manufacturer.

### Total-RNA extraction and quantitative reverse transcription-PCR

Total RNA was extracted using RNAiso Plus TRIzol (TaKaRa, Japan) according to the manufacturer’s manual. The RNA concentration was determined using a BioTeke spectrophotometer. One microgram of total RNA was used for cDNA synthesis using PrimeScript™ RT Master Mix (TaKaRa). TB Green™ Premix Ex Taq™ II (TaKaRa) was used as the fluorescent dye. All the primer pairs used in this experiment are shown in **Table S1**. The synthesized cDNA was subjected to qRT-PCR analysis using gene-specific primers. The mean fold-change of each gene was calculated and analyzed relative to controls by using the 2^−△△Ct^ method as described previously (Qin et al., 2013). All the assays were performed using an ABI 7300 system (Applied Biosystems, Massachusetts, USA).

### Western blot analysis

Western blot analysis was performed as previously described, but with some modifications (Qin et al., 2004; Qin et al., 2007). Briefly, cells were lysed on ice by using RIPA lysis buffer (Beyotime, China) containing a protease inhibitor cocktail and 1% phenylmethylsulfonyl fluoride (PMSF). Total protein concentrations of the supernatants were determined using the Bradford method (Beyotime). Subsequently, proteins were separated using 12.5% (w/v) SDS-polyacrylamide gel electrophoresis (PAGE) and transferred onto PVDF membranes (Millipore, USA) by using a Trans-Blot apparatus (Bio-Rad). The proteins of interest were identified using the indicated primary antibodies, and detected visually using horseradish peroxidase (HRP)-conjugated species-specific secondary antibodies. Immunoreactivity was visualized by enhanced chemiluminescence technique using SuperSignal West Pico chemiluminescent substrate (Thermo, USA).

### LysoSensor Green DND-189 staining

Lysosomal pH in Huh7 cells was determined using LysoSensor Green DND-189 (Invitrogen, L7535). Briefly, cells were infected with ZIKV at an MOI of 0.5 for 24 h or mock-treated. Next, they were incubated for 5 min at 37°C with 1 μM LysoSensor Green DND-189 diluted in pre-warmed complete medium. Afterward, they were washed twice with PBS and then provided with fresh medium. Finally, the fluorescence of the samples was quantified using a BioTek Synergy™ Mx Microplate Reader with 485-nm excitation and 530-nm emission filters.

### Statistical analysis

The data are presented as the mean ± SD from three independent experiments. Statistical significance of the differences was assessed using the Student’s *t*-test and GraphPad Prism software, and *p* < 0.05 (*), *p* < 0.01 (**), and *p* < 0.001 (***) were considered to indicate statistical significance.

## Results

### ZIKV infection induces lipophagy in Huh7 cells

**Figure 1:**
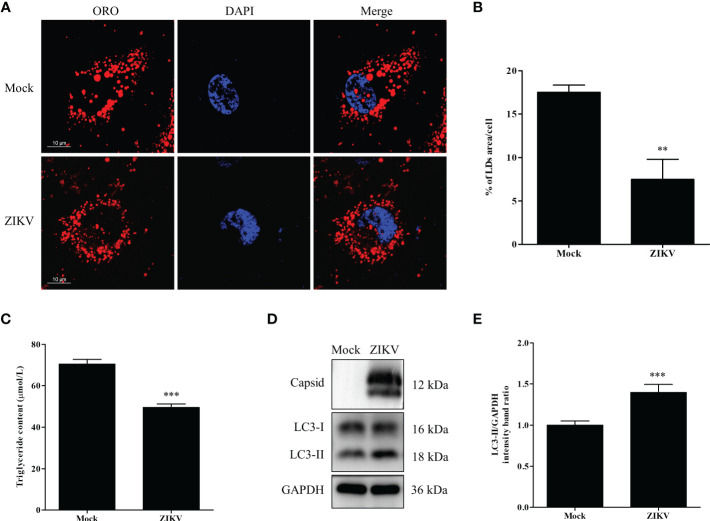
ZIKV infection induces lipophagy in human carcinoma cells. **(A)** Huh7 cells were infected with ZIKV at an MOI of 1 or mock-treated for 24 h. The cells were fixed, stained with Oil Red O (ORO), and then observed under a confocal microscope. Bars, 10 µm. **(B)** Quantification of the ORO-positive area per cell was performed using Image J. **(C)** The intracellular triglyceride content in mock-treated or infected cells was quantitated using a triglyceride assay kit. **(D)** Western blot analysis of viral Capsid and LC3 protein levels. GAPDH was used as a loading control. The turnover of LC3-I to LC3-II was assessed in cells infected with ZIKV at MOI 0.5 or mock-treated cells. Cells were harvested at 24 h and stained with corresponding antibodies. Representative images are presented. **(E)** The intensity band ratio of LC3-II to GAPDH, as measured *via* densitometry. Data are presented as the mean ± SD from three independent experiments. **p < 0.01, ***p < 0.001.

To determine whether lipid metabolism is altered by ZIKV infection, Huh7 cells were infected with ZIKV at an MOI of 1 or mock-treated for 24 h. The cells were then stained with the neutral lipid dye ORO to examine LDs under a confocal microscope. Compared with the mock-infected cells, the LD number noticeably decreased in the cytoplasm of the ZIKV-infected cells. Moreover, the average LD size in the ZIKV-infected cells was smaller than that in uninfected cells (**Figure 1A**). Quantitative analysis indicated that the LD area per cell and the TG level in the ZIKV-infected cells significantly decreased compared with those of the mock-infected cells (**Figures 1B, C**), indicating that lipid depletion and accumulation in cells are significantly increased and decreased during ZIKV infection, respectively.

Lipophagy is one of the essential pathways that activates degradation of cytosolic LDs. As an autophagic indicator, LC3, especially its membrane anchoring form (LC3-II), is critical for the formation of the double-membrane autophagosomes. Therefore, the conversion of endogenous LC3-I to LC3-II in cells infected with ZIKV at an MOI of 0.5 was assessed *via* western blot analysis 24 h after the infection. As demonstrated in **Figure 1D**, the ZIKV Capsid protein was used as evidence of ZIKV infection. The conversion of LC3-I to LC3-II, and LC3-II expression were increased in the infected cells, compared with the levels in mock-treated cells. To quantify the results, the intensities of the protein bands on the western blot were measured *via* densitometry analysis. The densitometry ratio of LC3-II to GAPDH was significantly increased in the ZIKV-infected cells 24 h after the infection, compared with the ratio in the mock-infected cells (**Figure 1E**). Collectively, these data indicate that ZIKV infection triggered cellular lipophagy in Huh7 cells.

### ZIKV infection upregulates lipophagy-related *HSL* gene in Huh7 cells

**Figure 2:**
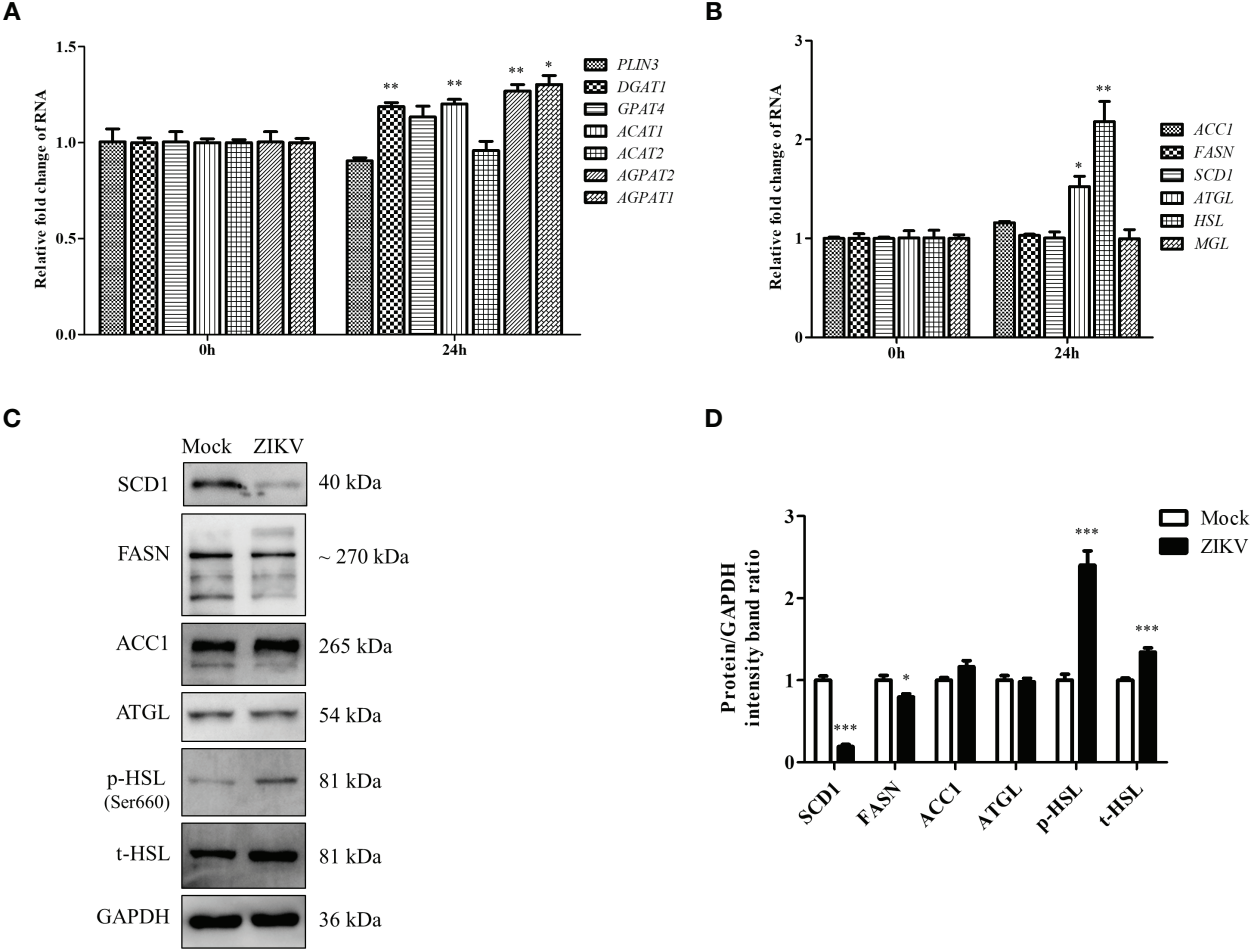
ZIKV infection promotes LD biogenesis and expression of lipolysis-related HSL gene in Huh7 cells. **(A–C)** Huh7 cells were infected with ZIKV at an MOI of 0.5 or mock-treated for 24 h. Samples were collected and subjected to qRT-PCR **(A, B)** or western blot analysis **(C)**. The mRNA levels of genes related to LD biogenesis **(A)**, lipid synthesis, or lipolysis **(B)** were determined. **(C)** Western blot analysis of SCD1, FASN, ACC1, ATGL, and HSL levels. GAPDH was used as a loading control. Representative images are presented. **(D)** The intensity band ratios of SCD1, FASN, ACC1, ATGL, p-HSL, and t-HSL to GAPDH were measured using densitometry. Data are presented as the mean ± SD from triplicate. *p < 0.05, **p < 0.01, ***p < 0.001.

The content of LDs in cells roughly reflects the balance between LD biosynthesis and degradation. LD biogenesis is regulated by various genes, including *PLIN3*, *DGAT1*, *GPAT4*, *ACAT1*, *ACAT2*, *AGPAT1*, and *AGPAT2* (Krahmer et al., 2009). The mRNA levels of these regulatory genes in cells infected with ZIKV at an MOI of 0.5 were measured *via* qRT-PCR 24 h after the infection. As shown in **Figure 2A**, *DGAT1*, *ACAT1*, *AGPAT1*, and *AGPAT2* mRNA levels were increased after ZIKV infection (albeit < 1.5-fold compared with the levels in the mock control), indicating that ZIKV infection promotes LD biosynthesis. In addition to genes involved in LD biosynthesis, those involved in lipid synthesis and lipolysis can also affect LD homeostasis. Thus, the mRNA levels of lipid-synthesis genes (acetyl-CoA carboxylase 1 [*ACC1*], fatty acid synthase [*FASN*], and stearoyl-CoA desaturase 1 [*SCD1*]) and lypolysis genes (triglyceride lipase [*ATGL*], hormone-sensitive lipase [*HSL*], and monoacylglycerol lipase [*MGL*]) were measured using qRT-PCR. ZIKV infection had no significant effects on the genes involved in fatty-acid synthesis. However, the mRNA levels of the *ATGL* and *HSL* genes, which are associated with lipolysis, were significantly increased (1.5- and 2.2-fold, respectively, compared with the mock levels) (**Figure 2B**). We then measured ACC1, FASN, SCD1, ATGL, and HSL protein levels by using western blot analysis. Interestingly, obvious decreases in SCD1 and FASN levels were observed 24 h after ZIKV infection, compared with the mock levels, but no significant change in ACC1 or ATGL level was observed. Additionally, the levels of total HSL and Ser660-phosphorylated HSL were increased (**Figures 2C, D**), indicating that lipolysis is promoted during ZIKV infection. Taken together, these data suggested that ZIKV infection promoted lipolysis by upregulating HSL and thus enhanced lipophagy in Huh7 cells.

### ZIKV infection promotes LD-lysosome fusion in Huh7 cells

**Figure 3:**
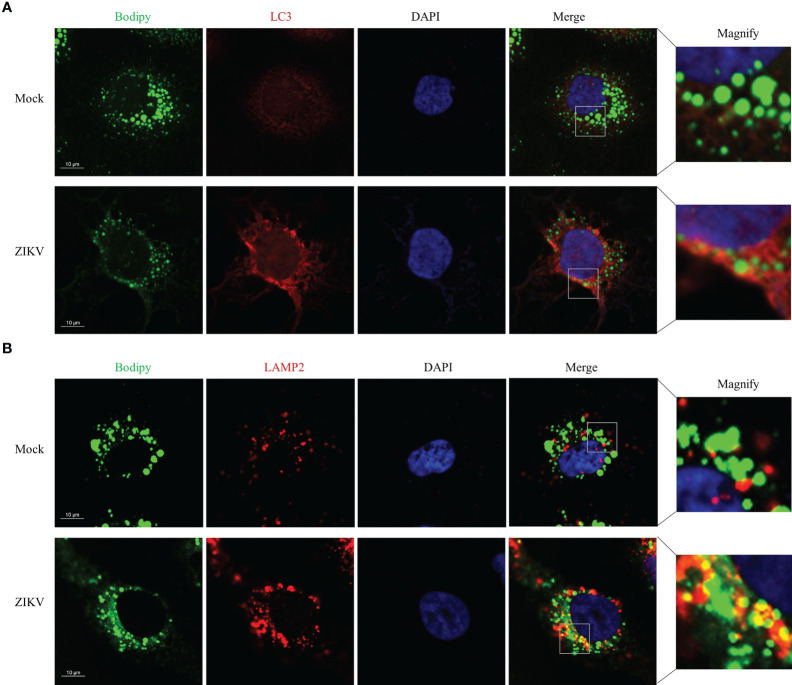
ZIKV infection Promotes LD-lysosome fusion in Huh7 cells. Huh7 cells were infected with ZIKV at an MOI of 1 or mock-treated for 24 h. The cells were fixed, stained with BODIPY dye or immunostained for LC3B **(A)** or LAMP2 **(B)**. Co-localization of LD with LC3-II or LAMP2 was assessed *via* confocal microscopy. Representative images are presented. Bars, 10 µm.

To determine how ZIKV infection enhanced lipophagy, we first characterized the subcellular distribution of LC3 and assessed whether LC3 co-localized with LDs. Toward this end, we co-stained ZIKV-infected cells with the BODIPY dye and LC3B immunostaining. We found that LC3-II was significantly upregulated and formed obvious aggregates upon ZIKV infection (**Figure 3A**). To test whether ZIKV infection promotes fusion of LDs with lysosomes, we assessed whether LDs co-localized with lysosome-associated membrane protein 2 (LAMP2), *via* confocal microscopy. More LDs co-localized with lysosomes in the ZIKV-infected cells than in the mock-treated cells (**Figure 3B**), indicating that LD-lysosome fusion significantly increased upon ZIKV infection. Furthermore, the small LDs seemed more evenly distributed, and thus lysosomes were presumably more accessible, in the ZIKV-infected cells than in the mock-infected cells. These data demonstrate that ZIKV infection promotes lipophagy, especially the fusion of LDs with lysosomes, in Huh7 cells.

### ZIKV infection promotes lysosomal biogenesis in Huh7 cells

**Figure 4:**
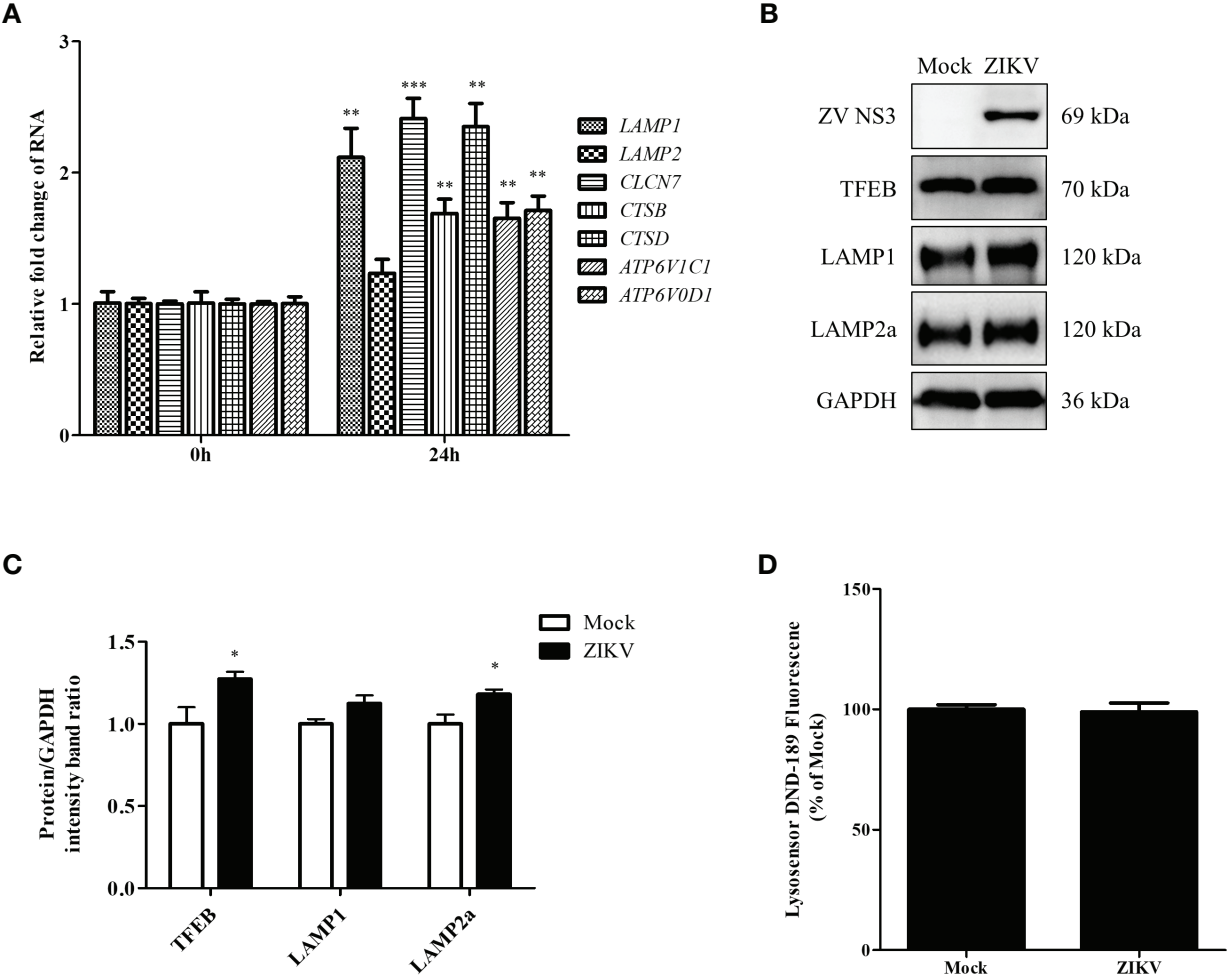
ZIKV infection promotes lysosomal biogenesis in Huh7 cells. Huh7 cells were infected with ZIKV at an MOI of 0.5 or mock-treated. Samples were collected at 24 h after ZIKV infection and subjected to qRT-PCR **(A)** or western blot analysis **(B)**. **(A)** The mRNA levels of lysosomal biosynthesis genes were examined. **(B)** Western blot analysis for TFEB, LAMP1, and LAMP2a levels. GAPDH was used as a loading control. **(C)** The intensity band ratios of TFEB, LAMP1, and LAMP2a to GAPDH were measured using densitometry. **(D)** The LysoSensor DND-189 fluorescence intensity in Huh7 cells was measured 24 h after ZIKV infection. All the experiments were repeated at least three times. Data are presented as the mean ± SD from triplicates. *p < 0.05, **p < 0.01, ***p < 0.001.

The last step of lipophagy is the degradation of LDs by lysosomes. Proper lysosomal function is critical for recycling LD contents. Enhanced lysosomal function may promote lipophagic flux. To further determine whether ZIKV infection affects lysosomal function, we first examined the mRNA levels of genes involved in lysosomal biosynthesis by using qRT-PCR. As shown in **Figure 4A**, ZIKV infection significantly upregulated genes related to lysosomal biosynthesis, including *LAMP1* and *CLCN7* (lysosomal transmembrane proteins); *CSTB* and *CSTD* (lysosomal hydrolases); and *ATP6V1C1* and *ATP6V0D1* (v-ATPase proteins). We also analyzed TFEB, LAMP1, and LAMP2a protein levels *via* western blotting. The ZIKV NS3 protein was used as evidence of ZIKV infection. ZIKV infection increased the levels of TFEB, which is a key regulator of lysosomal biogenesis and promotes autophagosome-lysosome fusion. The level of LAMP2a protein increased after ZIKV infection as well (**Figures 4B, C**), suggesting that ZIKV infection increased the number of lysosomes. LysoSensor Green DND-189 dye is an acid tropic probe that usually accumulates in acidic organelles, such as lysosomes, and exhibits a pH-dependent increase in fluorescence intensity upon acidification (Raben et al., 2009). No significant change on the fluorescence of this dye was observed after ZIKV infection (**Figure 4D**), indicating that ZIKV infection did not alter lysosomal pH in Huh7 cells. Together, the above results demonstrate that ZIKV infection induces the complete lipophagic flux by promoting lysosomal biogenesis and function in Huh7 cells.

### ZIKV infection activates the AMPK/ULK1 and AMPK/mTOR/ULK1 signaling pathways in Huh7 cells

**Figure 5:**
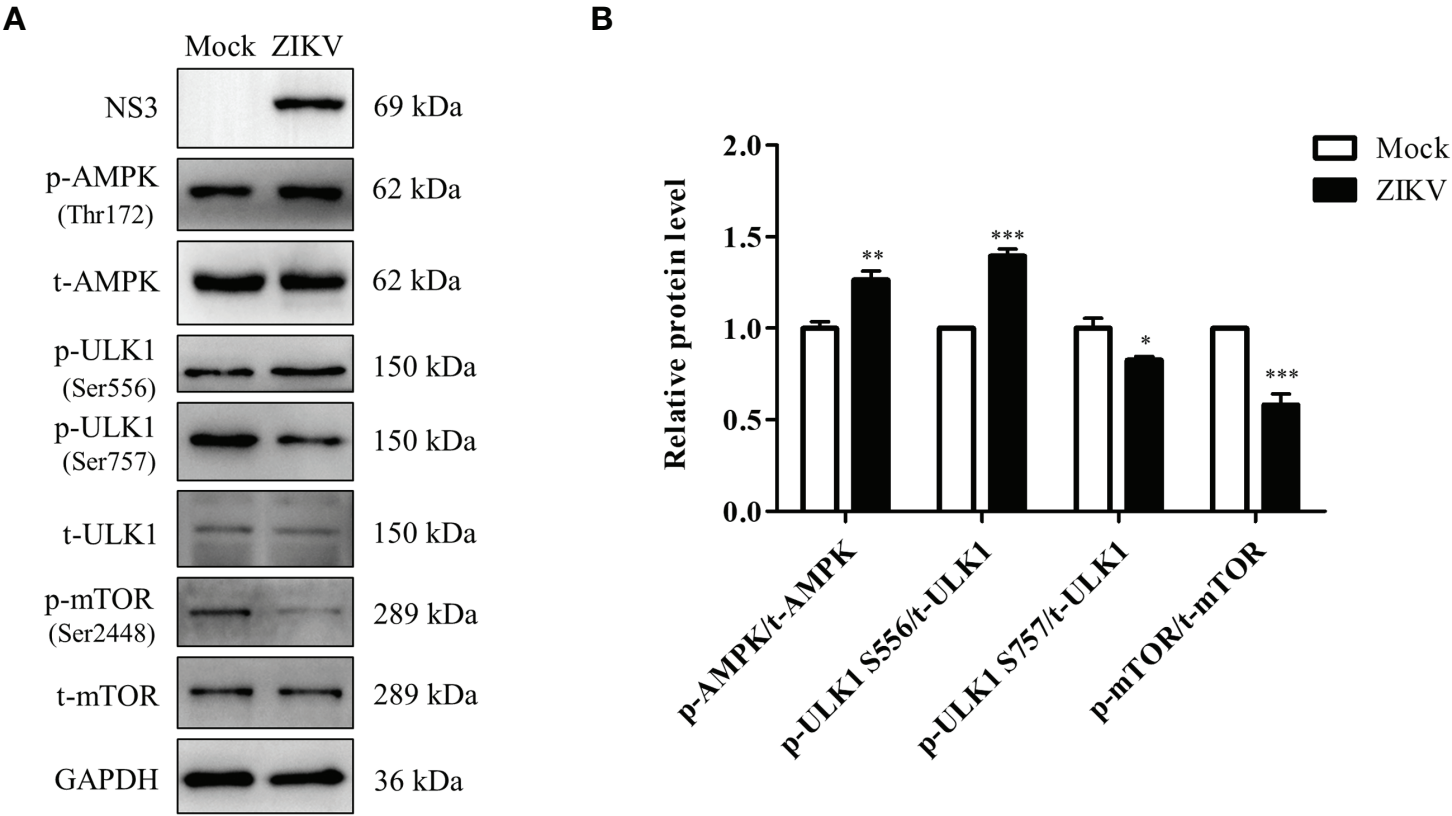
ZIKV infection activates the AMPK/ULK1 and AMPK/mTOR/ULK1 signaling pathways in Huh7 cells. Huh7 were infected with ZIKV at an MOI of 0.5 or mock-treated. Samples were collected at 24 h after ZIKV infection and subjected to western blot analysis. **(A)** Western blot analysis of the levels of AMPK, ULK1, mTOR, and their corresponding phosphorylated forms. GAPDH was used as a loading control. **(B)** The intensity band ratios of phosphorylated AMPK, ULK1, and mTOR to respective total protein levels were measured using densitometry. Data are presented as the mean ± SD from triplicates. *p < 0.05, **p < 0.01, ***p < 0.001.

Regulation of lipophagy relies on integration of multiple factors regulating autophagy and lipolysis. The AMPK and mTOR signaling pathways are the two essential cellular energy-sensing pathways that affect both autophagy and lipophagy (Zhang, X. Y. et al., 2018). ULK1 is located at the intersection of these two pathways and plays different roles depending on its phosphorylation at different sites, such as Ser556 and Ser757. To evaluate if ZIKV infection modulates the two pathways, the levels of the ULK1, AMPK, and mTOR proteins and their phosphorylated forms in ZIKV-infected Huh7 cells were measured using western blotting. As shown in **Figure 5A**, the ZIKV NS3 protein was used as evidence of ZIKV infection. Compared with the levels in mock-infected cells, ZIKV infection decreased the level of total AMPK but increased the level of Thr172–phosphorylated AMPK (AMPKαT172). Additionally, the levels of Ser556–phosphorylated ULK1 and Ser757–phosphorylated ULK1 were increased and decreased, respectively, and no significant change in the level of total ULK1 was observed. The level of Ser2448–phosphorylated mTOR in the infected cells was much lower than that in the mock-treated cells, but the level of total mTOR was not affected (**Figure 5A**). The increased ratio of the level of phosphorylated AMPK to total AMPK level 24 h after the infection indicated the ZIKV-induced increase in AMPK activation. Likewise, the ratio of Ser556–phosphorylated ULK1 to total ULK1 was also increased after ZIKV infection, whereas the ratios of Ser2448–phosphorylated mTOR to total mTOR and Ser757–phosphorylated ULK1 to total ULK1 were decreased (**Figure 5B**). These results demonstrate that ZIKV infection increased the activity of AMPK and Ser-556 phosphorylation of ULK1, but decreased the activity of mTOR and Ser-757 phosphorylation of ULK1. Accordingly, the AMPK-dependent lipophagy induced by ZIKV might be regulated by two signaling pathways—the AMPK/ULK1 and AMPK/mTOR/ULK1 pathways.

### Knock-down of AMPK expression inhibits ZIKV infection and lipophagy in Huh7 cells

**Figure 6:**
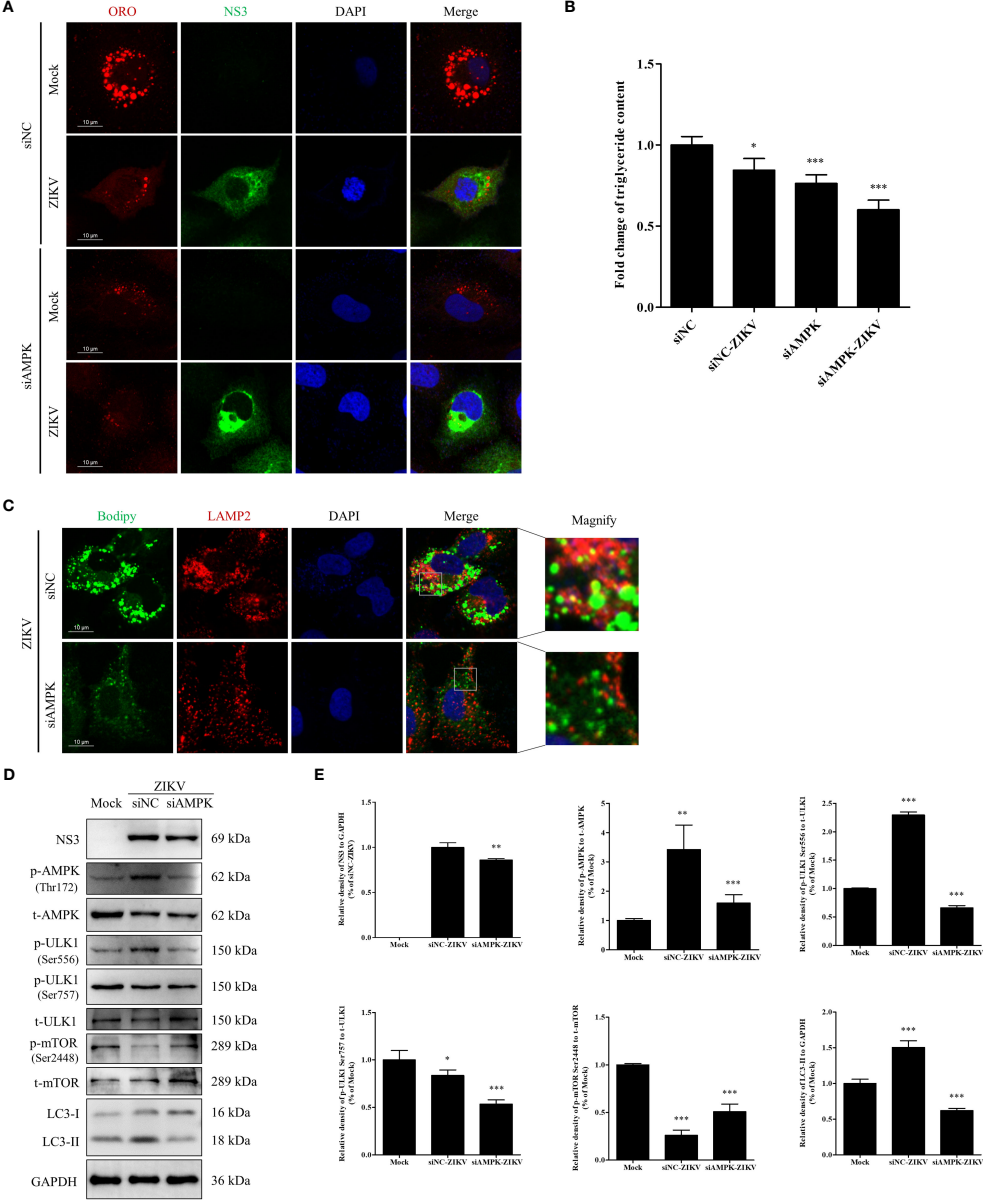
Knock-down of AMPK expression inhibits ZIKV infection and lipophagy in Huh7 cells. Huh7 cells were transfected with a scrambled negative control siRNA (siNC) or siAMPK for 48 h and then infected with ZIKV or mock-treated for another 24 h. **(A)** The cells were fixed, stained with Oil Red O, and ZIKV NS3 antibody and then observed under a confocal microscope. **(B)** The intracellular triglyceride content in the mock-treated or infected cells was quantitated using a triglyceride assay kit. **(C)** The cells were fixed, stained with BODIPY, and LAMP2 antibody and then observed *via* confocal microscopy. **(D)** Western blot analysis to assess the levels of AMPK, ULK1, mTOR, and their corresponding phosphorylated forms. GAPDH was used as a loading control. **(E)** The intensity band ratios of phosphorylated forms of AMPK, ULK1, and mTOR to respective total protein levels were measured using densitometry. Data are presented as the mean ± SD from three independent experiments. Bars, 10 µm. *p < 0.05, **p < 0.01, ***p < 0.001.

To further confirm whether AMPK-mediated signaling is required for ZIKV infection to induce lipophagy and mobilization of LDs, we transfected cells with an siRNA against *AMPKα* to knockdown AMPKα expression or with siNC as a negative control. In the siNC-treated cells, ZIKV infection significantly decreased the number and size of LDs (**Figure 6A**), consistent with the results shown in **Figure 1A**, indicating that LDs were depleted upon ZIKV infection. AMPK silencing significantly reduced the number and size of LDs in both mock- and ZIKV-infected cells, and fewer LDs were observed in the infected cells (**Figure 6A**). Quantitation of cellular TG indicated that the TG level was decreased by approximately 16%, 24% and 40% in the siNC-ZIKV, siAMPK, and siAMPK-ZIKV groups, respectively, compared with the level in siNC-treated cells (**Figure 6B**). This observation suggests that AMPK plays a critical role in the maintenance of cellular LD reservoir. We next assessed whether LDs co-localized with LAMP2 in cells transfected with siNC or siAMPK followed by ZIKV infection. As shown in **Figure 6C**, AMPK silencing significantly reduced the number of cellular LDs and the co-localization of LDs with lysosomes, indicating reduced lipophagy in infected siAMPK-treated cells.

The effects of AMPK silencing on ZIKV infection and the ULK1 and mTOR signaling pathways, two pathways downstream of AMPK, were evaluated *via* western blotting. Mock-infected cells were used as negative control. Compared with the siNC-treated cells, AMPK silencing downregulated viral NS3 and LC3-II, suggesting that AMPK silencing inhibited ZIKV infection and also autophagy. Compared with the level in the mock-treated cells, the level of total AMPK was significantly reduced in both siNC- and siAMPK-treated cells after ZIKV infection, whereas the level of phosphorylated AMPK in the siAMPK-treated cells was much lower than that in the siNC-treated cells. The ratio of Ser556-phosphorylated ULK1 to total ULK1 significantly decreased in infected siAMPK-treated cells. The ratio of Ser2448–phosphorylated mTOR to total mTOR was higher in the siAMPK-treated cells than that in the siNC-treated cells but still lower than that in the mock-infected cells.

Surprisingly, the ratio of Ser757-phosphorylated ULK1 to total ULK1 in the infected siAMPK-treated cells was much lower than that in the siNC-treated group, which is inconsistent with the change in mTOR activity (**Figures 6D, E**). Taken together, these data indicate that AMPK-mediated signaling is required for ZIKV-induced lipophagy. Accordingly, the AMPK/ULK1 signaling, not the AMPK/mTOR/ULK1 signaling, is involved in both autophagosome formation and mobilization of LDs in ZIKV-infected cells.

## Discussion

Lipophagy, a selective autophagy targeting LDs, is another mechanism for removing cellular lipids in addition to lipolysis. In this study, we demonstrated that ZIKV infection triggered lipophagy in Huh7 cells, as evidenced by increased autophagy alongside decreased LD-positive area and TG level. ZIKV-induced lipophagy appears mainly regulated by the AMPK-ULK1-Ser556 signaling pathway.

LD is a specialized organelle for energy storage in most types of cells. With changes in cellular metabolism, the size and number of LDs in the cytoplasm can dynamically change through formation and expansion or contraction and lysis (Brasaemle and Wolins, 2012). Large LDs are usually regarded as a more efficient form of fat storage than small LDs. Therefore, to some extent, LD size can reflect the rate parameters of lipid storage and lipid mobilization. In this study, LDs became smaller, and their TG content decreased upon ZIKV infection. Both LC3-II level and colocalization of small LDs with lysosomal LAMP2a were significantly increased in the infected cells. This observation is consistent with previous findings that small LDs are more amenable for lipophagic internalization than larger LDs (Schott et al., 2019). These data suggest that ZIKV-induced lipophagy promotes lipid mobilization in LD to meet the energy demands during the viral replication.

Small LDs appear to provide more area for lipases and subsequent metabolism of cellular lipids. Lipases involved in the degradation of the TGs in LDs include ATGL and lysosomal lipase. ATGL is recruited to LDs, and its efficient access to LDs is an initial step to promote TG catabolism, which is followed by sequential catalyzation by HSL (hydrolyzes diglycerides) and MGL (hydrolyzes monoglycerides) (Fredrikson et al., 1986; Smirnova et al., 2006; Ong et al., 2011). The expression of lysosomal lipase is regulated by TFEB, which was significantly upregulated upon ZIKV infection. *ATGL* and *HSL* mRNA levels were increased; especially, the *HSL* mRNA was increased by > 2-fold 24 h after ZIKV infection. Correspondingly, both total and Ser660-phosphorylated HSL levels were significantly increased, indicating increased TG degradation. Surprisingly, ATGL protein level was not significantly increased upon ZIKV infection. ATGL promotes lipophagy *via* SIRT1 activity to control the catabolism of hepatic LDs (Sathyanarayan et al., 2017). The LIR domain of ATGL enables the interaction of ATGL with LC3. This interaction promotes ATGL translocation to LDs and then induces lipophagy. Additionally, the level of ATGL, as the first rate-limiting enzyme in TG lipolysis, is often proportional to the rate of lipolysis (Chakrabarti et al., 2011). ATGL has a short half-life and is degraded by the ubiquitin-proteasome system, and this regulatory mechanism has been shown to affect ATGL content and TG turnover in the liver (Dai et al., 2013; Ghosh et al., 2016). Thus, our results suggest that ATGL undergoes a post-translational modification and participates in both lipolysis and lipophagy during ZIKV infection.

LDs are multifunctional dynamic organelles, and their abnormal metabolism is closely linked to the etiology of various cases of dyslipidemia (Greenberg et al., 2011). Cell signaling pathways, such as the AMPK signaling, play an important role in the regulation of lipophagy. To assess the activity of AMPK during ZIKV infection, we examined AMPK activity in cell lysates 24 h after ZIKV infection. Compared with the levels in uninfected cells, the total level of AMPK was decreased during ZIKV infection, whereas Thr172 phosphorylation of AMPK, indicative of AMPK activity, was significantly increased. Consistent with this observation, ZIKV infection decreased mTOR activity and enhanced autophagy. Moreover, ZIKV infection increased Ser556 phosphorylation of ULK1 and significantly reduced Ser2448 phosphorylation of mTOR and Ser757 phosphorylation of ULK1. These results indicate that AMPK activation and mTOR inhibition together activate ZIKV-induced lipophagy.

Interestingly, AMPK activation is also involved in the maintenance of cellular lipid homeostasis by inhibiting the key enzymes in the lipid anabolic pathway, such as ACC1 and FASN (Chukkapalli et al., 2012; Huang et al., 2019). During Rift Valley Fever Virus infection, AMPK is activated and then inhibits viral replication by inactivating ACC1 to limit fatty-acid synthesis (Moser et al., 2012). Additionally, AMPK can regulate cholesterol synthesis through the direct phosphorylation of HMGCR and thereby affect intracellular lipid content (Soto-Acosta et al., 2017). Although the expression of the *DGAT1*, *ACAT1*, *AGPAT2*, and *AGPAT1* genes, which are involved in LD biosynthesis, was activated, fatty-acid synthases (SCD1 and FASN, but not ACC1) were significantly downregulated during ZIKV infection. Nevertheless, these observations cannot exclude the possibility that increased AMPK activity inhibits the expression of SCD1 and FASN through a similar mechanism to temporarily limit viral infection after ZIKV infection. Taken together, our results suggest that ZIKV sequentially activates the catabolic metabolism (AMPK-activated lipophagy) and anabolic metabolism (fatty-acid synthesis, even though its key enzymes are inhibited) pathways. Nevertheless, viral-infection–induced lipophagy (reduced LD-positive area due to lipophagy) and fatty-acid biosynthesis may be segregated and coordinated spatially (e.g., in different subcellular compartments) and temporally (e.g., at different stages of infection), thereby creating favorable conditions for the viral replication.

In ZIKV-infected cells, AMPK inhibition significantly reduced LD accumulation (significant reduction in ORO area), autophagosome formation (decreased LC3-II protein level), and ZIKV NS3 protein level, suggesting that the lipophagy inhibition caused by AMPK knockdown blocks the viral replication. A previous study has shown that inhibition of AMPK expression or kinase activity blocked DENV replication and production of infectious virus particles mainly because the AMPK silencing prevented the depletion of LDs in DENV-infected cells (Jordan and Randall, 2017). The reason why AMPK silencing causes different effects on LDs in Huh7 versus HepG2 cells needs further exploration. Although AMPK knockdown has been shown to block the infection of ZIKV and DENV, the inhibitory rate of AMPK knockdown on the expression of the ZIKV NS3 protein was estimated at approximately 14% in this study, suggesting that there may be AMPK-independent pathways required for ZIKV infection. Notably, decreases in LD-positive area and TG level were also observed in siAMPK-treated uninfected cells, suggesting that AMPK knockdown blocks lipophagy by reducing the number of LDs and the size of the LD-positive area.

The ULK1 activity in AMPK-mediated autophagy pathways is regulated through various post-translational modifications. Activated AMPK regulates autophagy either through the mTOR-ULK1 pathway, which promotes autophagosome biogenesis, or by directly binding to and phosphorylating the ULK1/2 complex to drive autophagy independently of mTOR (Cloherty et al., 2020). ULK1 is phosphorylated at Ser757 and Ser556 in the two pathways, respectively. In this study, increased phosphorylation of ULK1-Ser556 and reduced phosphorylation of mTOR-Ser2448 and ULK1-Ser757 were observed along with increased AMPK activity after ZIKV infection. When AMPK was knocked down, the level of Ser556-phosphorylated ULK1 was decreased as expected, and although the level of Ser2448-phosphorylated mTOR was increased, it was still lower than the level in the siNC control group, suggesting that some other factors may inhibit mTOR activation during ZIKV infection. With the enhanced inhibition of mTOR activity, the level of Ser757-phosphorylated ULK1 did not increase but instead decreased. Additionally, decreased LC3-II level was observed in the siAMPK-treated cells after ZIKV infection. Together, these data indicate the importance of AMPK activity in autophagy and lipophagy. mTOR inhibition is not the only pathway that activates autophagy. Compared with the complex mTOR-ULK1-Ser757 pathway, the direct Ser556 phosphorylation of ULK1 by AMPK is simpler in regulating ZIKV-induced lipophagy. The precise regulation of the AMPK-mTOR-ULK1-Ser757 pathway needs to be further investigated in ZIKV-infected siAMPK-treated cells.

Activation of lipophagy is restricted to a specific environment and closely related to LDs and energy demands required by the environment. Rapid utilization of lipid reserves or elimination of excess lipids appear to be an evolutionary mechanism for cells to maintain lipid homeostasis. However, the mechanism of lipophagy regulation, especially how autophagy selectively recognizes and sequesters LDs, remains largely unknown. Thus, it is necessary to identify additional key proteins and signaling networks in lipophagy.

In conclusion, we found that ZIKV infection induces AMPK-mediated lipophagy in Huh7 cells. AMPK and its downstream ULK1-Ser556 signaling pathway are centrally involved in the regulation of the LD metabolism required for ZIKV infection. Accordingly, this study provides new insights into the mechanism of the lipophagy in ZIKV infection.

## Data availability statement

The original contributions presented in the study are included in the article/**Supplementary Material.** Further inquiries can be directed to the corresponding authors.

## Author contributions

Conceptualization: ZLQ and ZTQ. Investigation, Data curation: ZLQ and QFY. Methodology: ZLQ. Formal analysis: ZLQ and QFY. Validation, supervision: ZLQ, PZ, HR and ZTQ. Writing-original draft: ZLQ. Writing-review and editing: ZLQ, PZ, HR and ZTQ. All authors contributed to the article and approved the submitted version.

## Conflict of interest

The authors declare that the research was conducted in the absence of any commercial or financial relationships that could be construed as a potential conflict of interest.

## Publisher’s note

All claims expressed in this article are solely those of the authors and do not necessarily represent those of their affiliated organizations, or those of the publisher, the editors and the reviewers. Any product that may be evaluated in this article, or claim that may be made by its manufacturer, is not guaranteed or endorsed by the publisher.
