# Supplementary material for: Zika virus infection triggers lipophagy by stimulating the AMPK-ULK1 signaling in human hepatoma cells
Source: Front Cell Infect Microbiol. 2022 Nov 2;12:959029. doi: 10.3389/fcimb.2022.959029 (PMC9667116; doi:10.3389/fcimb.2022.959029)
Supplement: Supplementary file 1 [file Table_1.doc]

**Table S1. Sequences of primers used for quantitative real-time PCR**

| Target genes | primers |
| --- | --- |
| PLIN3 | F: 5’-CAGCCCACGGAGAAGGTGTC-3’  R: 5’-GCCGGTCACTACGGACTTTG-3’ |
| DGAT1 | F: 5’-AACAAGGACGGAGACGCC-3’  R: 5’-TCAGGATGCCACGGTAGTTG-3’ |
| GPAT4 | F: 5’- ATTGTCTGCAGCGTGTGGTA -3’  R: 5’- CTGCTCCTCCTTGAACGTGT -3’ |
| ACAT1 | F: 5’-GAAGCCTTTAGTCTGGTTGTACT-3’  R: 5’- TCCTGGCTCCAGACATCCTAAA -3’ |
| ACAT2 | F: 5’- AAAAGCAGGTTGGTCACTGG -3’  R: 5’- CGACTTCTGCCCATTCTCTC -3’ |
| AGPAT1 | F: 5’- TGGCCTTCTACAATGGCTGG -3’  R: 5’- ACAACAACATAGGGCTGCGA -3’ |
| AGPAT2 | F: 5’- GAGGGTACTCGCAACGACAA -3’  R: 5’- CCTGCACTGTGACTGTTCCT -3’ |
| ACC-1 | F: 5’- TAGTCTGCCACGGATCCAGA -3’  R: 5’- GGGAGGGATCTCTGAGGGTT -3’ |
| FASN | F: 5’- CCTGGCTGCCTACTACATCG -3’  R: 5’- CACATTTCAAAGGCCACGCA -3’ |
| SCD1 | F: 5’- CCACTTGCTGCAGGACGATA -3’  R: 5’- CGTCTTCCAAGTAGAGGGGC -3’ |
| ATGL | F: 5’-CCAATGTCTGCAGCGGTTTC-3’  R: 5’-GACAGATGTCACTCTCGCCC-3’ |
| HSL | F: 5’- TCAGACTGGCAACCTGAACC -3’  R: 5’- TCATGTTGTGCAGGGGTCTC -3’ |
| MGL | F: 5’- TTGCTGCGAAAGTGCTCAAC -3’  R: 5’- ATTCAGCAGTTGGATGCCGA -3’ |
| LAMP1 | F: 5’- CTGCCTTTAAAGCTGCCAAC -3’  R: 5’- TGTTCTCGTCCAGCAGACAC -3’ |
| LAMP2 | F: 5’- GGTTAATGGCTCCGTTTTCA -3’  R: 5’- ATGGGCACAAGGAAGTTGTC -3’ |
| CLCN7 | F: 5’- GGGATCTCTCAGGGAAGGTC -3’  R: 5’- AATTCAGGGTGAACGTGGAG -3’ |
| CTSB | F: 5’- GGAGGGAGATACCCCCAAGT -3’  R: 5’- ACGGGGCCGTTTTTGTAGAT -3’ |
| CTSD | F: 5’- GGCGAGTACATGATCCCCTG -3’  R: 5’- CGACACCTTGAGCGTGTAGT -3’ |
| ATP6V1C1 | F: 5’- GCATGCGGCAACTTCAAAGA -3’  R: 5’- GCCAGTTCATCTGACAAGCC -3’ |
| ATP6V0D1 | F: 5’- GTCGTTCTTCCCGGAGCTTT -3’  R: 5’- CACCAGGTTGAGGTAGTCGG -3’ |
